# Serious Game Development for Public Health: Participatory Design Approach to COVID-19 Quarantine Policy Education

**DOI:** 10.2196/54968

**Published:** 2024-10-15

**Authors:** Myunghwan Kwak, Byeong-Je Kim, Ji-Bum Chung

**Affiliations:** 1 Department of Civil Urban Earth and Environmental Engineering, Ulsan National Institute of Science and Technology, 50, UNIST-gil, Ulsan, 44919, Republic of Korea, 82 010-2488-1506; 2 The Institute of Social Data Science, Pohang University of Science and Technology, Pohang-si, Gyeongsangbuk-do, Republic of Korea; 3 Division of Advanced Nuclear Engineering, Pohang University of Science and Technology, Pohang-si, Gyeongsangbuk-do, Republic of Korea

**Keywords:** online learning, serious game, simulation, quarantine policy, social distancing policy, game, public health education, infectious diseases, learner-centric

## Abstract

**Background:**

Public health education plays a crucial role in effectively addressing infectious diseases such as COVID-19. However, existing educational materials often provide only foundational information, and traditional group education faces challenges due to social distancing policies.

**Objective:**

Addressing these gaps, our study introduces a serious game called “Flattening the Curve.” This interactive experience immerses learners in the role of quarantine policy managers, offering unique insights into the effects and challenges of social distancing policies.

**Methods:**

The development of the game adhered to the SERES framework, ensuring a scientifically designed foundation. To achieve its learning objectives, the game incorporated learning and game mechanics including an agent-based infection model, a social distancing policy model, and an economic model, which were developed based on previous literature. After defining a broad concept of scientific and design foundations, we used a participatory design process. This study included 16 undergraduates and took place over one semester. Participants played the game, gave feedback, and answered surveys. The game was improved based on participants’ feedback throughout the process. Participants’ feedback was analyzed based on the Design, Play, and Experience framework. Surveys were conducted before and after the activity and analyzed to assess participants’ evaluation of and satisfaction with the game.

**Results:**

The game successfully achieved its learning objectives, encompassing a comprehensive understanding of infectious disease characteristics; the disease transmission process; the necessity and efficacy of quarantine policies and their delicate balance with economic factors; and the concept of flattening the curve. To achieve this, the game includes the following: (1) an agent-based infection model based on the modified Susceptible-Exposed-Infectious-Hospitalized-Recovered (SEIHR) model with five infectious disease scenarios; (2) a quarantine policy model with social distancing, travel control, and intensive care unit management; and (3) an economic model that allows users to consider the impact of quarantine policies on a community’s economy. In response to participatory design feedback, the game underwent meticulous modifications, including refining game systems, parameters, design elements, the user interface, and interactions. Key feedback included requests for more scenarios and engaging yet simple game elements, as well as suggestions for improving the scoring system and design features. Notably, concerns about the fairness of the outcome evaluation system (star rating system), which could incentivize prioritizing economic activity over minimizing casualties, were raised and addressed by replacing the star rating system with a progress-based vaccine development system. Quantitative evaluation results reflect participants’ positive assessments of the game through the learner-centric approach.

**Conclusions:**

The serious game “Flattening the Curve,” developed through a participatory design approach, emerges as a valuable tool for public health education, particularly concerning social distancing policies. The game and its source code are openly accessible online, enabling widespread use for research and educational purposes.

## Introduction

### Background

Since December 2019, the COVID-19 pandemic has profoundly impacted global society, affecting numerous domains such as daily life, health care, and education, among others. In an effort to mitigate the impacts of COVID-19, governments worldwide have implemented a range of social distancing measures, including compulsory mask wearing, curfews, stay-at-home orders, and lockdowns. However, the implementation of these policies has led individuals to experience economic, social, and psychological loss, resulting in diminished trust in their respective governments [1,2]. This sense of loss and eroded trust are fueling public rejection of social distancing policies and even leading to active defiance of governments. Indeed, several countries experienced increased public protests due to the social distancing policies [3-5].

People’s noncompliance with social distancing policies can be attributed to various reasons. Previous research suggests that these reasons include a lack of knowledge about infectious disease and health, misunderstanding, and misinformation about mitigation policies [6-9]. To effectively prevent and control the transmission of infectious diseases such as COVID-19, adherence to public health policies, including quarantine measures, is crucial. Additionally, fostering public comprehension of the necessity and rationale underpinning these policies is of paramount importance [3].

To achieve these objectives, effective communication with the public and the provision of education aimed at enhancing the general public’s understanding of policy validity and scientific knowledge are necessary. Currently, educational resources pertaining to infectious diseases predominantly focus on health-related information and individual behaviors, while the development of materials designed to foster comprehension of national- or community-level social distancing policies remains insufficient. Moreover, the majority of training materials employ a unilateral instructional approach, relying on simplistic documents, images, and videos. These conventional methods generally prove inadequate in fostering public empathy toward the policy-related challenges confronted by quarantine officers. On the other hand, game-based learning using serious games is known to increase education effectiveness and learning motivation through the interactive and participatory nature of games [10-12].

Due to the COVID-19 pandemic, the demand for health and safety education has increased, and many researchers have developed and used serious games for COVID-19–related education. The primary education topics include COVID-19 knowledge [13-15] or infection prevention behaviors and perceptions [10,15-18]. Since serious games are suitable for distance education and e-learning (posing less of a concern in terms of infection), they are an educational method that has been in the spotlight for many researchers in the pandemic era, coinciding with an increase in online education [19-22].

Earlier studies found that game-based learning could result in higher knowledge acquisition [12,23] and, more importantly, that games could make their players motivated and satisfied [11,23]. Digital game–based learning enabled by serious games can make a learning environment more active, engaging, and immersive, which is important for effective learning [24]. However, most game-based health and safety education focused on teaching safe behavior, knowledge, and adequate use of personal protective equipment to prevent COVID-19 infection. The transfer of mere knowledge has limitations in imparting an understanding of why compliance with quarantine policies is necessary and how the policies work. Therefore, by using existing education, learners were not properly motivated to go beyond personal safety behaviors and comply with quarantine policies. A serious game for quarantine policy education will be a fundamental driving force for the public to learn why the social distancing policy must be followed rather than just how to comply with it.

### Objective

The objective of this study was to address a critical gap in existing educational resources for quarantine policies by developing an engaging and interactive learning tool. To achieve this objective, the game incorporates scientifically designed game-based learning mechanics including an agent-based infection model, a social distancing policy model, and an economic model. Furthermore, a participatory design (PD) process was conducted, with this learner-centric approach enhancing the educational effectiveness of the game. This study endeavors to enhance public understanding of quarantine policies by disseminating the developed serious game through web platforms, thereby promoting informed decision-making and adherence to quarantine measures.

## Methods

### General Study Design

To effectively deliver quarantine policy education addressing infectious diseases to the public, we developed a serious game named “Flattening the Curve” (FTC). The serious game used in this study was designed in a form that could motivate learners’ interest while conveying scientific facts. The game was developed using the SERES framework [25]. The SERES framework is a 5-step framework for developing health care serious games. It has been applied to the development of various health care serious games and gamified e-learning materials.

In addition to using the SERES framework, the game was developed through a PD process to enhance the engagement and motivation of the learners [26,27]. PD is a research method in which users directly or indirectly participate in the design process and compose and interpret the design together with designers, researchers, and developers [28,29]. From an educational point of view, PD promotes learners’ active engagement through motivation and internalization of concepts [30,31].

The design process was conducted as part of the undergraduate disaster management course at the Ulsan National Institute of Science and Technology, which was taught by the corresponding author during the second semester of 2021. Participants participated from the 2nd to the 15th week of the semester. To recruit participants, the authors explained to the participants the study’s purpose, overall process, and tasks to be performed. The authors offered them the opportunity to participate in this activity as part of their coursework. All students voluntarily agreed to participate in the study.

This PD process reflected the iterative game design process, including design, prototype, and playtest [32]. Participants’ comments and suggestions were analyzed and applied to the game using the 4 layers of the Design, Play, and Experience (DPE) framework [33]. During the PD process, participants answered two surveys, in the 3rd week and 14th week, respectively. Participants’ key feedback and related implementation are presented in the Results section, and the details of the survey instrument are also presented in Multimedia Appendix 1.

### Ethical Considerations

This study was reviewed by the Institutional Review Board at the Ulsan National Institute of Science and Technology and was determined to be exempt (UNISTIRB-21‐58C). Due to the COVID-19 pandemic and the shift to remote learning, obtaining written informed consent was difficult. Therefore, verbal consent was obtained from all participants prior to their involvement in the study. All collected data were deidentified. There was no penalty for students who did not participate in part or all of the activity. Participants did not receive monetary compensation. However, to encourage active participation, participants who performed well in the activity were awarded extra credit for the course. This compensation was announced after all students had agreed to participate in the study, to ensure that the incentive did not coerce participation.

### Scientific Foundations

The game was developed for people of all ages to facilitate an understanding of quarantine (social distancing) policies. Emphasis was placed on ensuring that the in-game explanations are easily comprehensible for players of all ages.

**Figure 1.**
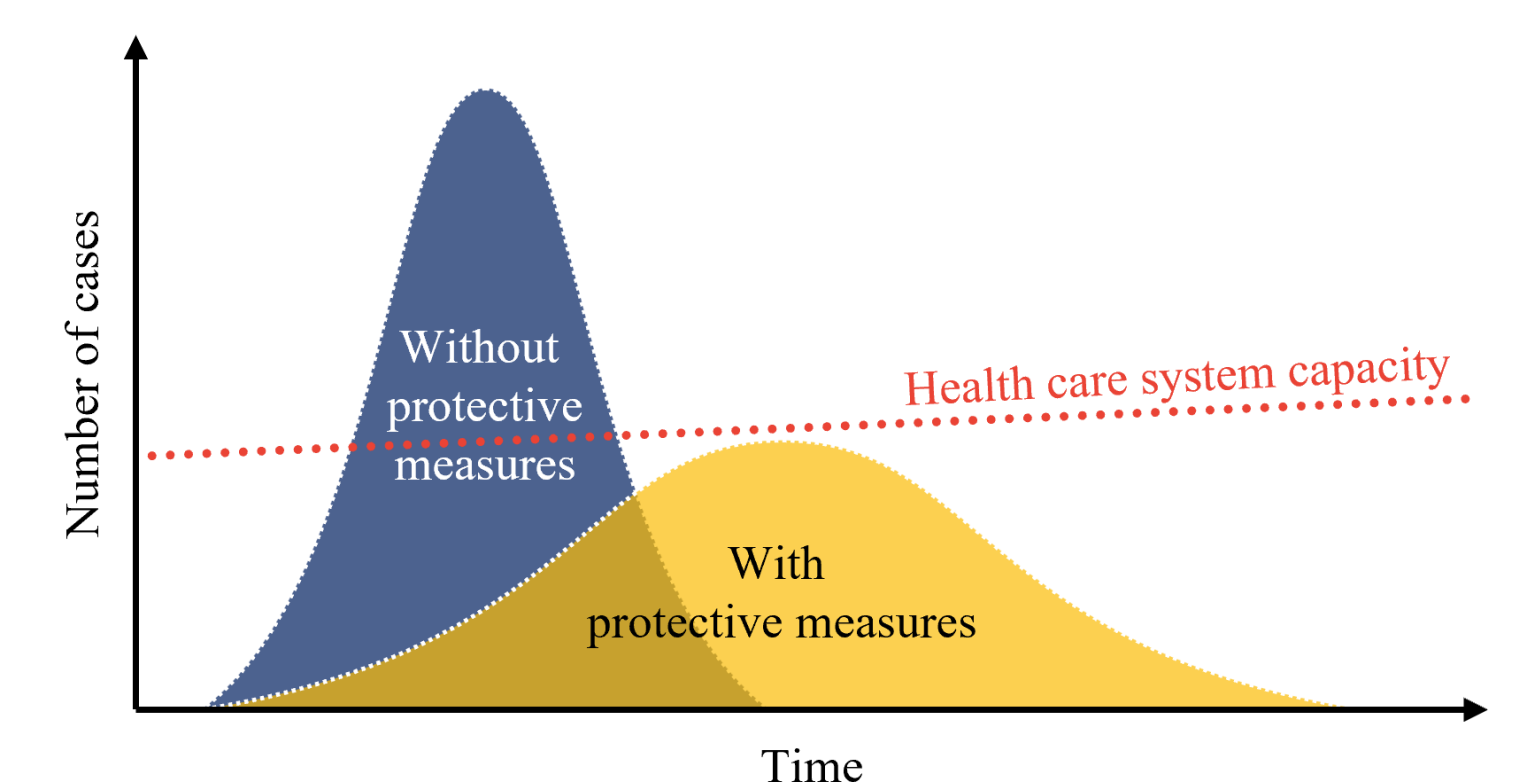
An illustration of the concept of “flattening the curve.” Slowing the spread of infection is essential to ensure that people have easier access to treatment, while also allowing time to gradually increase the capacity of the health system.

Understanding how and why a policy is important can lead to behavior changes in learners [34,35]. According to theories of human behavior related to safety and health, such as the Health Belief Model, the perceived benefits expected from engaging in certain behaviors can impact an individual’s behaviors [36]. In other words, a thorough understanding of how quarantine policy benefits individuals and our society can influence an individual’s compliance with policies. Thus, the primary learning objective of the game was for users to develop an understanding of the core concept of quarantine policies—“flattening the curve”—and recognizing the need for a balanced adjustment between preventive measures and economic consequences. Figure 1 illustrates the concept of flattening the curve, demonstrating how implementing social distancing measures can flatten the curve and consequently reduce the fatality rate [37]. In the absence of proper protective measures, the number of patients could easily overwhelm the health care system’s capacity, resulting in inadequate treatment for some. Therefore, the point of flattening the curve is to ensure that more patients receive proper treatment, ultimately reducing the fatality rate [38]. Within the simulation, changes in the transmission pattern of infectious diseases can be observed according to policy implementation.

**Figure 2.**
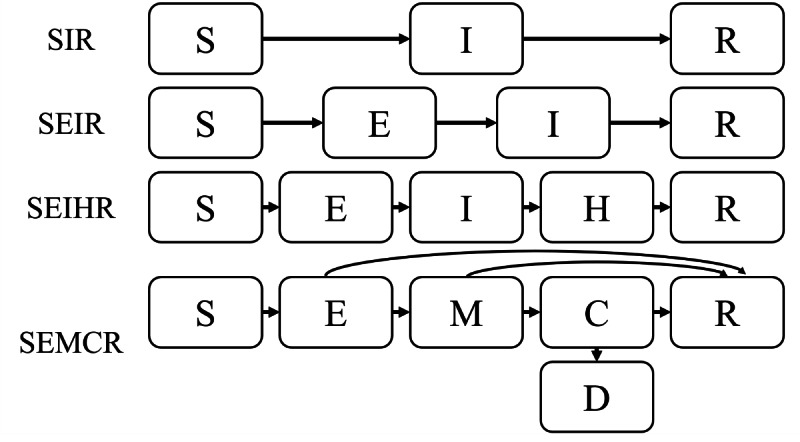
Flowcharts of epidemic models, edited from previous studies [39-41]. C: critical; D: dead; E: exposed or latent period; H: hospitalized; I: infectious; M: mild; R: recovered; S: susceptible.

The game mechanics should be designed based on proper scientific foundations. Several models were suggested to predict the spread of the epidemic and the effect of social distancing policy [39-41] (Figure 2). Kermack and McKendrick [42] first proposed the Susceptible-Infectious-Recovered (SIR) model to explain the spread of infections transmitted from person to person through mathematical approaches. SIR is the most fundamental infectious disease model, consisting of Susceptible, Infectious, and Recovered or Removed compartments. Anderson and May [43] introduced the SEIR model, which includes an additional Exposed condition (latent period or incubation period). SEIHR is a model that extends the SEIR model by incorporating an isolated (Hospitalized) condition [40]. Further, Yang et al [41] suggested another modified version of SEIR: SEMCR, which divided the infectious stage into mild (M) and critical (C) cases. Patients in the critical stage have a higher probability of death.

In the FTC game, we applied a modified epidemic model by integrating SEIHR [40] and SEMCR models [41] to distinguish between mild and critical cases and also include the hospitalized condition.

Based on the modified epidemic model, the game was designed to model various well-known infectious diseases, such as COVID-19 (Delta and Omicron variants), measles, and Ebola virus. Major characteristics of diseases, such as reproduction number and mortality rate, were adjusted based on statistical data [44-50]. Characteristics of COVID-19 variants were based on statistics provided by the Korea Disease Control and Prevention Agency [51]. The implemented infectious disease transmission model has been consulted and verified by a medical expert.

Social distancing is a public health strategy that aims to reduce the spread of infectious diseases [52]. It involves maintaining physical distance from others, avoiding large gatherings, and minimizing contact with individuals who are sick.

Researchers have employed various methods to implement social distancing in epidemic models. Social distancing is typically achieved by increasing the physical distance between people or reducing the number of physical contacts between individuals [41,53,54]. Agarwal et al [55] utilized daily movement rates to predict the number of deaths per day to evaluate the effectiveness of COVID-19 social distancing policies. Miksch et al [53] used location factors to make social distancing decrease the number of visitors to specific places such as schools and workplaces.

However, many educational materials related to infectious diseases have not adequately addressed the economic damage caused by the infectious diseases and the corresponding quarantine policies. Various social, health, and sanitary policies to prevent the spread of infectious diseases have been found to bring about negative economic impacts [56]. In the case of the United States, government measures such as business closures and restrictions on economic activities to slow the spread of diseases led to a record high in unemployment claims [57]. Therefore, we built economic elements into the game to help players learn about the economic impacts of quarantine policies.

### Design Foundations

The general design of the serious game was established according to Winn’s DPE framework [33]. At the initial stage of game development, we decided what types of learning and game mechanics should be included in the game based on the previous literature [58-60]. The game genre was decided to be a simulation, as its learning mechanism focuses on constructivist learning principles [60], which posit that learning draws upon prior knowledge to alter existing schema [61]. Using simulations, students have more opportunities to learn by doing, discover interesting infectious disease problems, and gain indirect experience in dealing with public health issues in the real world [62].

Design requirements considered the platform and accessibility to potential learners. The game was developed using JavaScript and published through a web page to be accessible to anyone. Following the players’ feedback, visual enhancements were implemented to enhance the game’s appeal to a younger audience.

## Results

### Design of the Serious Game

The game was developed as a turn-based strategic simulation game, with each turn corresponding to one week. In our interactive serious game, learners become a quarantine policy manager of a community. At the end of each week, users are allowed to choose appropriate quarantine measures. The spread of the epidemic is visualized on the game screen during the following week, and on weekends, users can check the status of the epidemic, apply quarantine measures, and continue on to the next turn. Players are provided with a budget necessary for implementing quarantine policies, which replenishes at the end of each month (every 4 weeks). The budget amount depends on the level of economic activity, as indicated by gross domestic product (GDP).

The objective of the gameplay is to minimize both casualties and economic damage until vaccine development is complete. Since the vaccine development speed depends on both the number of healthy people and GDP, faster vaccine development can be an indicator of a successful quarantine response.

To slow down the spread of disease, players should develop their own response strategies with various policies. These policies can be adjusted or changed on a weekly basis and each has its own advantages in reducing the spread of the disease. However, it is important for players to find a cost-effective combination of policies, as some have drawbacks (eg, social distancing policies may hinder economic activity, while policies such as travel control or increasing intensive care unit [ICU] capacity may require significant economic investments).

**Figure 3.**
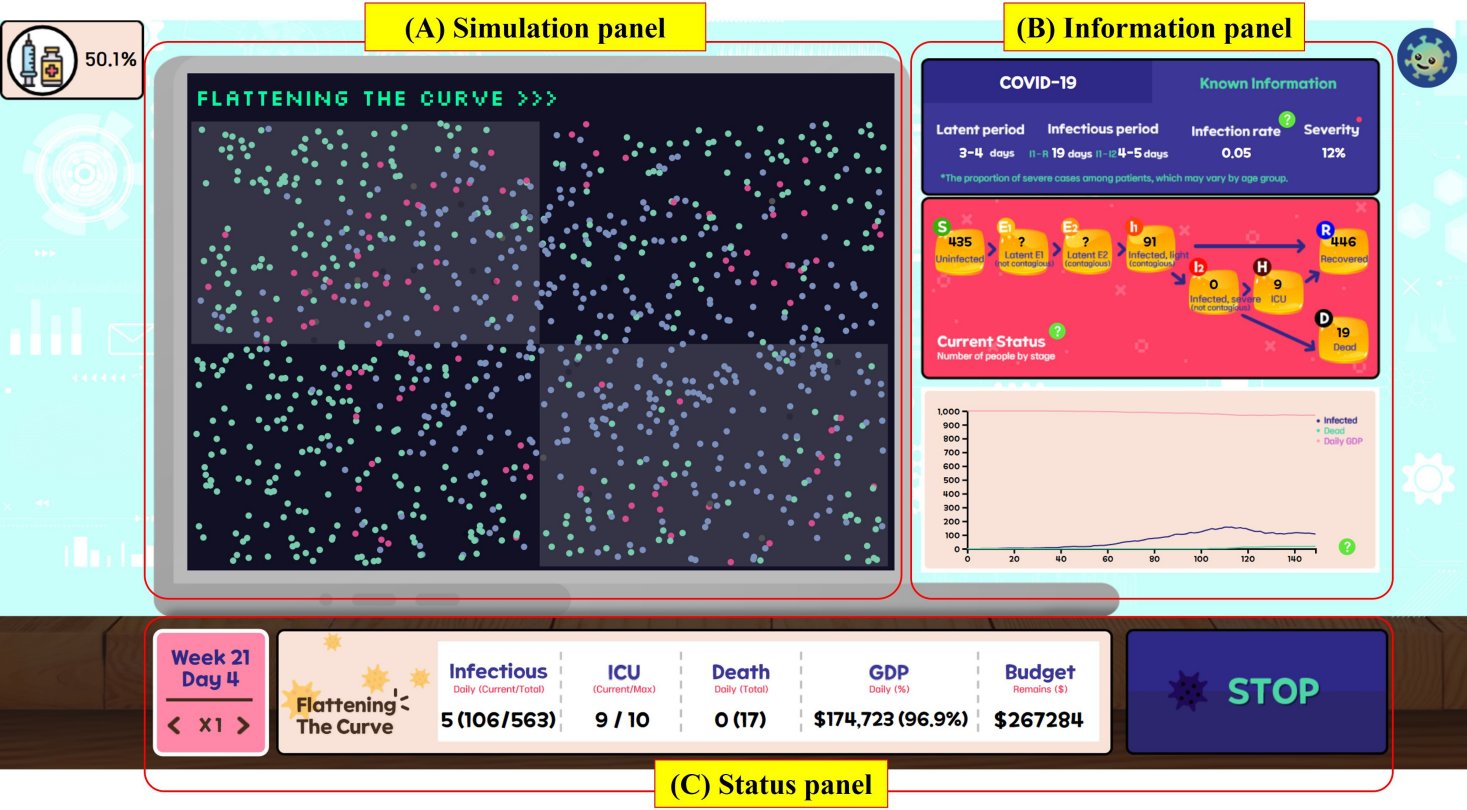
Interface of the final version of the Flattening the Curve game. (A) Simulation panel: visualizes the agent’s movements within the game environment. (B) Information panel: displays the current epidemic situation. At the end of each week, this panel also presents the weekly report. (C) Status panel: provides a summary of the key information. This panel also includes speed control and pause functions to allow players to adjust the pace of the game. GDP: gross domestic product; ICU: intensive care unit.

The game comprises 3 main panels: the simulation panel, information panel, and status panel (Figure 3). In the simulation panel, individuals are represented as round dots that move randomly. The color of each dot indicates its condition based on the modified SEIHR model. The information panel displays essential details about infectious diseases, such as the latent period, infectious period, infection rate, and severity. It also shows the number of people in each infectious stage, categorized according to the modified SEIHR model. The bottom of the panel features a graph illustrating overall disease trends.

**Figure 4.**
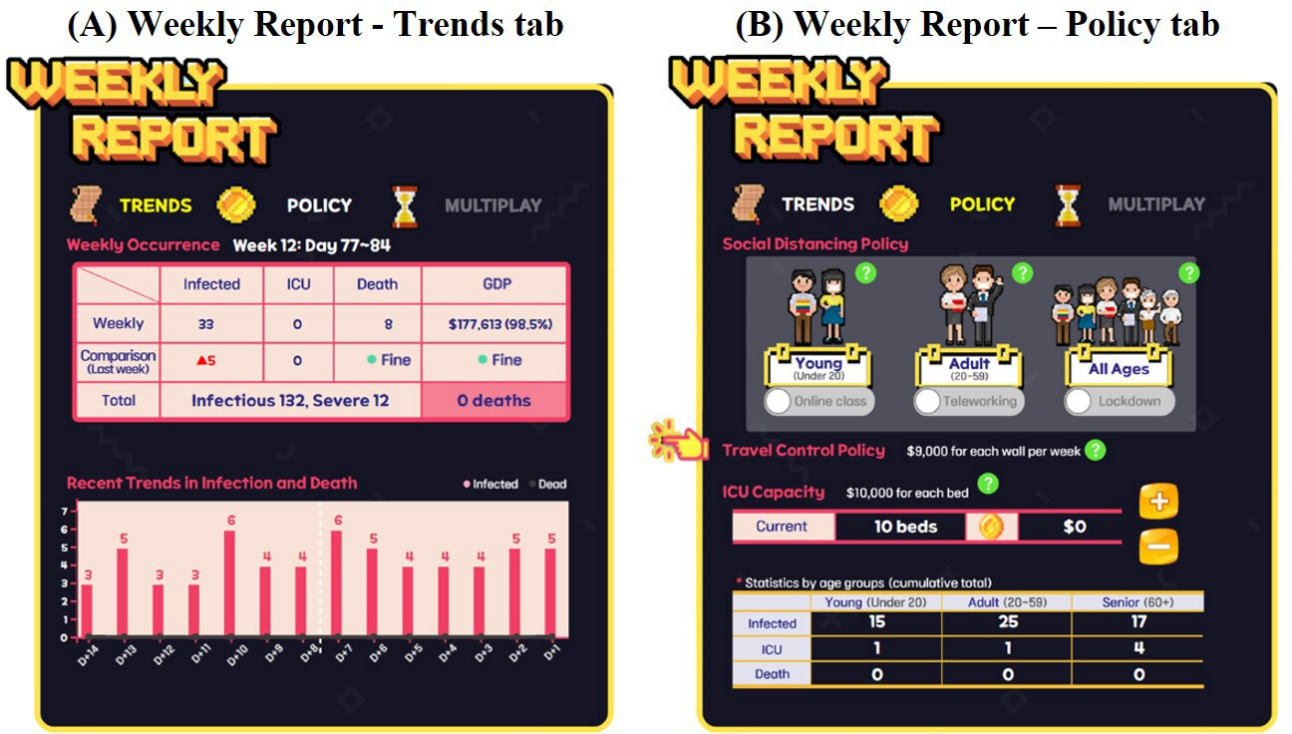
Subtabs in the weekly report panel. (A) Trends tab. (B) Policy tab. GDP: gross domestic product; ICU: intensive care unit.

At the end of each week, the information panel transforms into the weekly report panel (Figure 4). Within the weekly report panel, there are two subtabs: Trends and Policy. The Trends tab includes tables and graphs that present the number of infections, critical cases, and deaths, as well as GDP, for the current week, providing a comparison with the previous week. This feature assists users in understanding more nuanced infection trends. In the Policy tab, players can implement age-based social distancing policies, implement travel control measures, and enhance ICU capacity.

The status panel at the bottom consistently displays key information, including the current date, number of infections, ICU capacity, death toll, GDP, and budget status.

We made some important basic assumptions that make the game more explicit and learning oriented. These assumptions are carefully designed to ensure that the game retains its inherent learning capabilities and is sufficiently representative of the real world without becoming too complex. The assumptions are as follows.

1. Fixed population: there are 1000 people in the simulation at the start, with no population change due to death, birth, or migration outside of the epidemic.
2. Simplified interactions: a “contact” has occurred when dots overlap each other on the simulation panel.
3. Fixed border: the game world is assumed to have fixed rectangular boundaries and is divided into four in-game districts, whose geographic and social conditions are identical. This exists to determine the coverage of social distancing policies. There are no spatial features such as specific locations, workplaces, or homes.
4. Simplified economy: GDP is only influenced by how much people move around during the week. If social distancing policies are tightened or the number of deaths is high, people move around less, so the budget that the player receives decreases.
5. Simplified policy: quarantine policies, including the allocation of ICU beds, come into effect instantly, without any waiting or grace periods. In-game citizens do not raise objections to policies.

### Application of the Scientific Foundation

#### Overview

**Figure 5.**
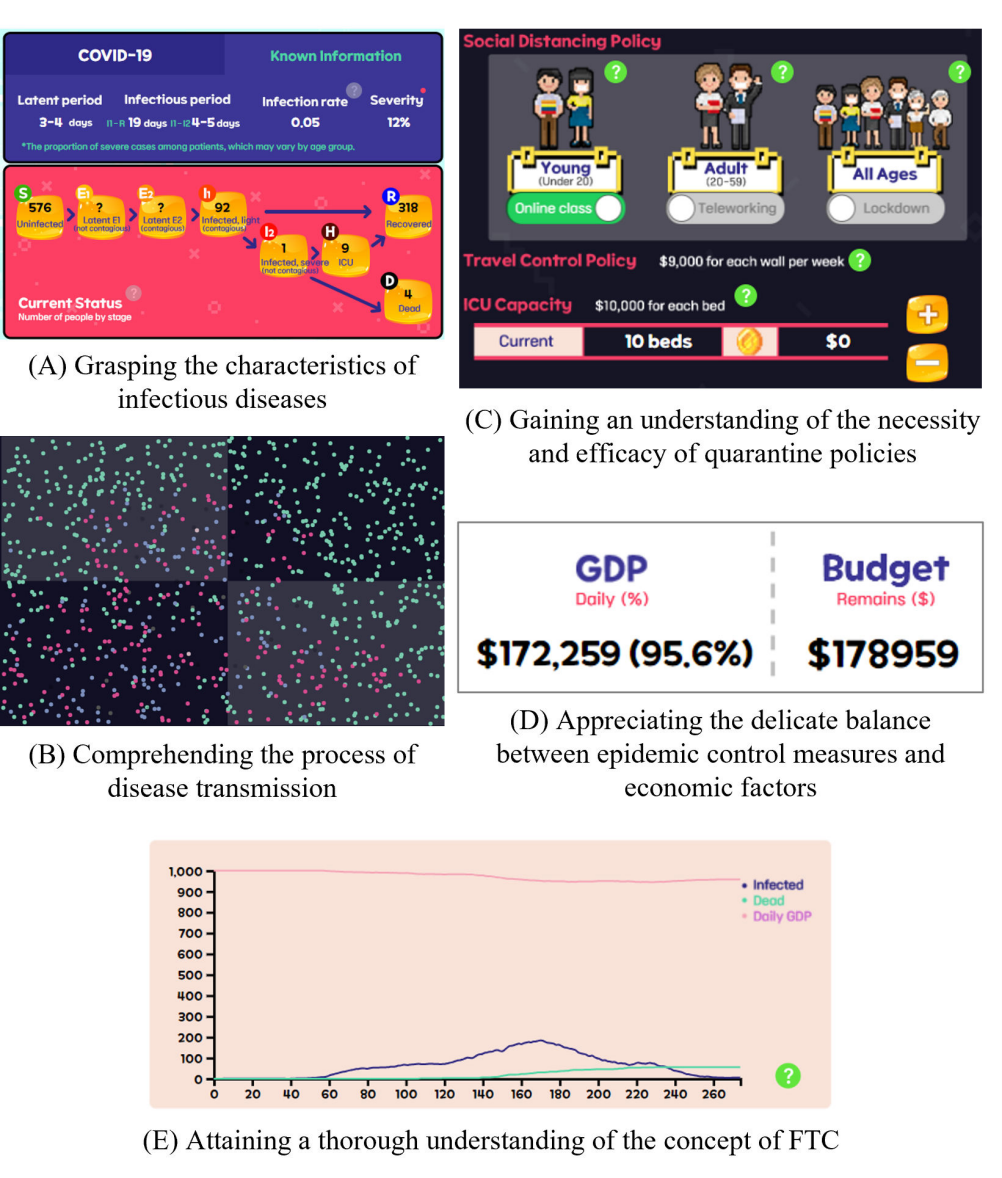
Learning objectives and corresponding game elements in the game. FTC: flattening the curve; GDP: gross domestic product; ICU: intensive care unit.

The ultimate objective of the game is to comprehend the concept of flattening the curve, necessitating an understanding of infectious disease characteristics and control policies. There are 5 learning objectives in the game to achieve this. Figure 5 demonstrates a visualization of the learning objectives covered in the developed game.

The first learning objective focuses on understanding the characteristics of infectious diseases. The game incorporates characteristics of infectious diseases, such as the incubation period, transmission probability, and fatality rate. With the modified SEIHR model, the game simulates detailed infection and progression pathways. This interactive approach allows learners to gain insights into the distinct features and stages of various infectious diseases through gameplay of various scenarios.

The second learning objective involves understanding the process of disease transmission within society. The game takes the form of a city management simulation, effectively portraying the process of disease transmission as people move within the city.

Building upon these objectives, the third learning objective emphasizes the necessity and efficacy of quarantine policies. During gameplay, learners are expected to actively participate in the implementation of quarantine policies in response to changing circumstances, thus developing a comprehensive understanding of the need for and effectiveness of policies.

The fourth learning objective addresses the balancing act between disease control policies and economic factors, illustrating the practical challenges of policy implementation. To convey the practical consequences of implementing quarantine policies, the game introduced economic concepts such as GDP and a budget, underscoring the imperative to strike a balance between epidemic control measures and economic considerations.

By building upon these 4 learning objectives, learners will eventually understand the concept of “flattening the curve” and gain the knowledge and skills to effectively manage infectious diseases.

All these learning objectives were delivered by scientifically designed game modules. The following sections describe how the modules were implemented in the game.

#### Infection Model

**Figure 6.**
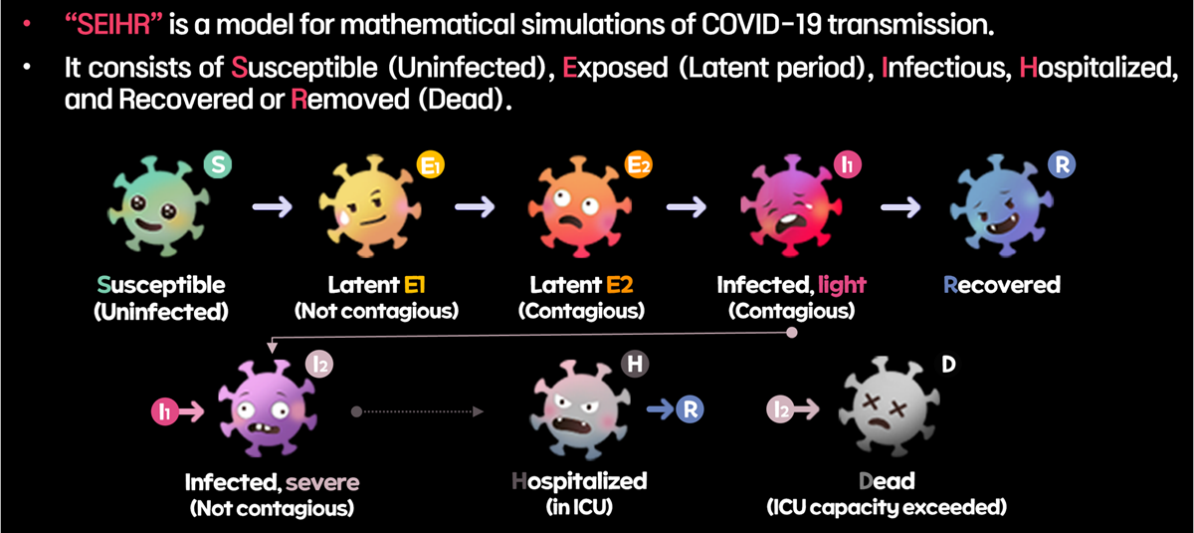
In-game description of the modified SEIHR model. ICU: intensive care unit; SEIHR: Susceptible-Exposed-Infectious-Hospitalized-Recovered.

The modified SEIHR model implemented in the game consists of distinct stages: Susceptible (S), Exposed (E, latent period), Infected (I), Hospitalized (H), Recovered (R), and Dead (D) (Figure 6). When a noninfected agent (S) contacts a symptomatic agent (E2 or I1), they enter the latent period (E) based on infection probability. After a certain duration, the infection progresses to symptomatic mild stage (I1).

In the earlier exposed phase (E1), agents do not have symptoms and are not infectious. Over time, they change to an exposed state with infectiousness (E2). Infected individuals are categorized into 2 groups based on the severity of symptoms. Mild patients (I1) can infect others and eventually recover (R) after a designated period. Some patients may advance to severe cases (I2) based on predefined probabilities linked to disease characteristics and age. If an ICU is available, severe patients can be isolated (H) and recover (R); if ICU isolation is not possible, these patients face mortality (D). The initial ICU bed capacity is limited and treatment in the ICU takes a considerable amount of time. Therefore, players are prompted to use their budget to expand ICU capacity. The ages and severity rates of agents in the game are distributed similarly to real-world statistics based on relevant data provided by the Korea Disease Control and Prevention Agency [51] (Table 1).

In addition, we have incorporated various infectious disease scenarios to facilitate a natural understanding of disease characteristics. Table 2 shows the latent and infection period, average infection rate, and morbidity of each disease implemented in the game. The included infectious diseases are COVID-19 and its variants (Delta and Omicron), measles, and the Ebola virus. This allows coverage of a spectrum of infectious diseases, ranging from those with low mortality rates and high infection rates to representative diseases with higher mortality rates and slower transmission.

**Table 1.** Population distribution and severe morbidity by age group (based on COVID-19, the default scenario).

	Age range (years)								
	0‐9	10‐19	20‐29	30‐39	40‐49	50‐59	60‐69	70‐79	≥80
Distribution, %	8	10	13	13	16	18	13	5	2
Morbidity, %	1.6	1.6	1.6	3.7	3.7	13.4	27	54	54

**Table 2.** Infectious disease scenarios and major characteristics.

Disease type	Latent period (days)	Infection period (days)		Infection rate, %	Morbidity, %
S to E1^a^	I1 to R^b^	I1 to I2^c^
COVID-19	3‐4	19	4‐5	5	12
COVID-19 (Delta variant)	3‐4	19	4‐5	13	14
COVID-19 (Omicron variant)	3‐4	19	4‐5	58	3
Ebola	1‐2	18	3‐4	2	55
Measles	3‐4	17	2‐3	17	0

a S to E1: Susceptible to Exposed (earlier, noninfectious stage).

b I1 to R: Infectious (mild case) to Recovered.

c I1 to I2: Infectious (mild case) to Infectious (critical case).

#### Quarantine Policy Model

Every 7 days, players can choose an appropriate quarantine policy based on the current status of disease spread. In the game, social distancing, travel control, and ICU expansion policies are implemented.

Social distancing policies are implemented based on the “containment and closure” category policies, according to the COVID-19 government response tracker from Oxford [63]. This category includes measures such as school closures, workplace shutdowns, cancellation of public events, restrictions on gatherings, public transport closures, stay-at-home orders, limitations on movement between cities or regions, and restrictions on international travel. As simplified by participants’ feedback, the game only incorporates school closures (affecting those under 20 years), workplace shutdowns (affecting those between 20 and 50 years), and lockdowns (affecting all age groups). Although social distancing policies effectively control the spread of infectious diseases, they inevitably reduce overall public mobility, leading to a decrease in economic activity. This can constrain budgets for other control measures and potentially slow vaccine development. Consequently, players must carefully consider the long-term trade-offs when implementing social distancing strategies.

With the travel control policy, players can build walls at the boundaries of selected areas to prevent travel across those boundaries. This effectively restricts people within a zone from traveling to another zone, but it requires a weekly budget to maintain the walls. Furthermore, the policy has been designed to not entirely restrict travel between zones in order to accommodate for the restrictions of the real-world travel control policy. As a result, the effectiveness of the policy may vary depending on the pattern of infection spread. This requires players to carefully consider the spatial distribution of infections and strategically implement this policy to optimize timing and location to maximize impact.

ICU expansion permanently increases the number of available ICU beds through budget spending. The concept of flattening the curve is implemented through this. Players should slow the spread of infectious diseases through quarantine measures while continuously investing in ICUs to increase medical capacity and reduce deaths [64]. Expanding ICU capacity is crucial but expensive. As disease transmission stabilizes and ICU demand declines, some additional beds may become underutilized. This highlights the need for players to consider the long-term cost-effectiveness of such investments.

#### Economic Model

Although quarantine policies prove effective in curtailing the spread of infectious diseases, they carry the side effect of impeding economic activities [56,57]. To enhance learners’ understanding of how quarantine policies impact a community’s economy, we integrated a GDP-based economic module into the game. Economic activity within the game was designed to be correlated with the movement speed of agents, setting GDP as the total accumulation of economic activities over a week (ie, the sum of movement distances). The movement speed of each agent is contingent on factors such as age group, quarantine policies, and the prevailing infection situation. The economically active population aged 20‐59 years moves at a faster pace than other age groups, with mild patients experiencing a 10% reduction in movement speed and severe patients being unable to move until recovery.

GDP functions as an indicator gauging the overall level of local economic activity, and players are allocated a budget each month proportional to the GDP of the preceding 4 weeks. This budget is essential for implementing travel control policies and expanding ICU capacity. Users face dilemmas, pondering whether to accept economic losses through stringent social distancing policies to slow the spread of infection or to mitigate economic damage through lenient social distancing policies while securing crucial budgets for augmenting ICU capacity.

### Game Development With PD

To enhance participants’ engagement and motivation when playing the serious game, we employed PD and analyzed learner feedback based on the DPE framework [33]. All 16 students in the class took part in the PD process. Throughout the activities, participants played the game, shared their experiences and opinions, and provided recommendations, which were mostly incorporated into the final version. Following the DPE framework, participants’ feedback was categorized according to subcomponents of serious game design: learning, storytelling, gameplay, and user experience (Table 3).

**Table 3.** Participants’ key feedback and related implementation examples.

User feedback by Design, Play, and Experience layers		In-game implementation
**Learning**		
	Users can understand the dynamics of disease spread through visual simulations. Learning about the differences in disease spread patterns based on virus characteristics. Understanding the necessity and effectiveness of various public health policies. Balancing adjustments are needed (eg, enhancing the effectiveness of social distancing policies).	Game parameters such as default movement speed, maximum impact of social distancing policies, and amount of budget were adjusted to achieve optimal game balance.
**Storytelling**		
	More scenarios are desired.	Implementation of various infectious diseases beyond COVID-19.
	Immersion in the narrative is hindered by the star rating system.	Addition of a vaccine development system to replace the scoring system.
**Gameplay**		
	New systems like locations and behavior patterns are needed.	Introduction of a 4-zone travel control system.
	There are too many elements requiring complex intervention.	Simplification of policy user interface.
**User experience**		
	User interface and design improvements are needed.	Design rework and implementation of a speed control feature.
	Lack of guidance on how to use the game (need for a tutorial) and need to add feedback elements like alerts and pop-ups.	Pop-ups for budget system and implementation of Key Facts.

In the learning component, participants generally agreed that the game enabled them to learn the intended learning objectives. However, in the initial version, feedback arose regarding the game’s balance, causing issues like uncontrollable spread of disease or overly strong social distancing effects. To address these problems and enhance learning, efforts were made to balance the game, including lowering default agent movement speed and reducing the maximum impact of social distancing policies on movement speed.

Regarding the storytelling component, even though there was no direct narrative or story in the game, participants immersed themselves in the game by adopting the background narrative of being the “public health manager.” Feedback suggested enhancing the narrative, leading to the introduction of various types of infectious disease scenarios that players could select at the beginning of the game.

In addition, the initial version of the game ended when the infectious disease was eradicated, and game results were rated on a 10-star scale based on cumulative confirmed cases (up to 1 star), deaths (up to 4), economic activity levels (up to 3), and remaining budget (up to 2). However, this star rating system impeded the players’ empathy and made gameplay time too long. Further, a fairness issue [65] arose as players prioritized economic activity over managing deaths to gain more stars. To solve these problems, a new clear condition (vaccine development) was introduced. The progress of vaccine development increases each week based on the number of survivors without severe symptoms and the amount of GDP. Players can expedite vaccine development by focusing on minimizing casualties and economic damage. The final version of the game ends when the vaccine development progress reaches 100% or a year passes.

**Figure 7.**
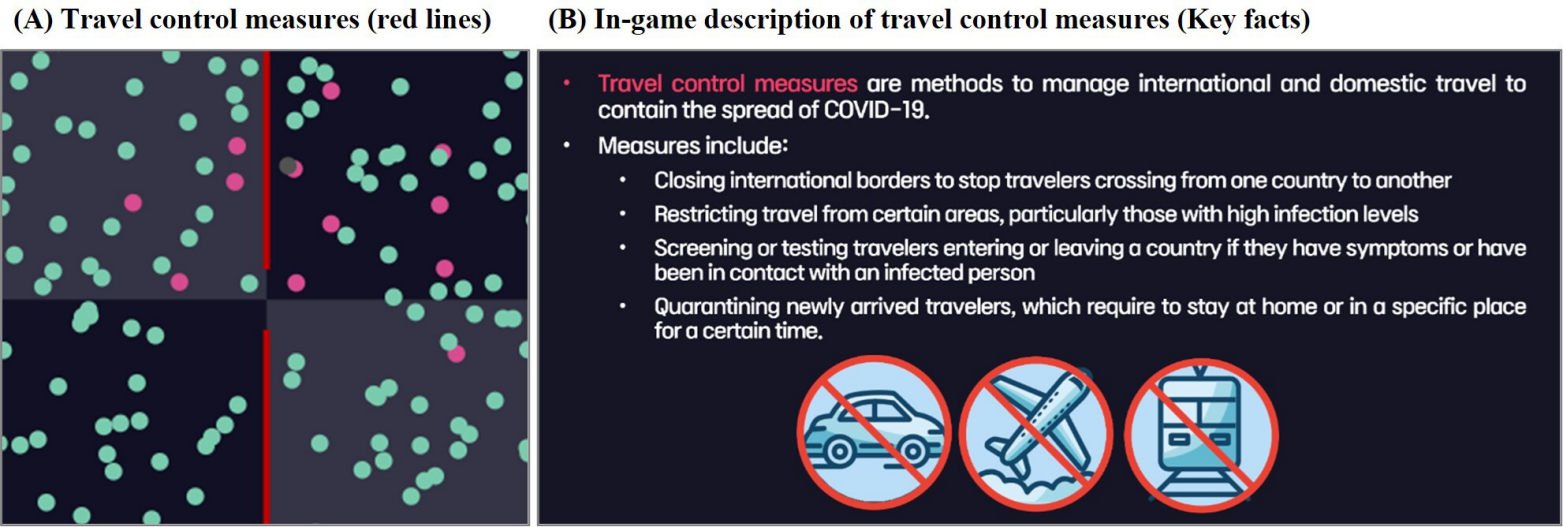
(A) The image illustrates the implementation of travel control measures, where red lines indicate blocked movement between zones. (B) An in-game description of travel control measures.

**Figure 8.**
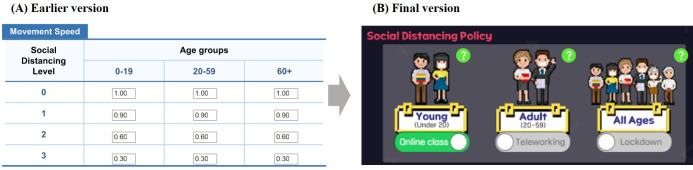
Example changes to improve UI for the social distancing policy. (A) The earlier version UI. (B) The final version UI. UI: user interface.

Regarding the gameplay components, participants mainly proposed new features such as events in new locations, gatherings, and activities in specific areas of the game. However, considering the potential excessive influence of these features on the game’s outcomes, seen as too random, we have introduced a new social distancing policy, the travel control system (Figure 7). In the later stages of the PD process, after more game features had been implemented, opinions regarding the simplification and improvement of existing features were added. For example, players were originally able to manually set the reduction in movement speed for each age group at each policy stage and activate the policies. However, as players perceived this feature to be too complex and began to underutilize it, we opted for a user interface (UI) enhancement, replacing the user customization feature with a simpler on/off mechanism for policy activation (Figure 8).

**Figure 9.**
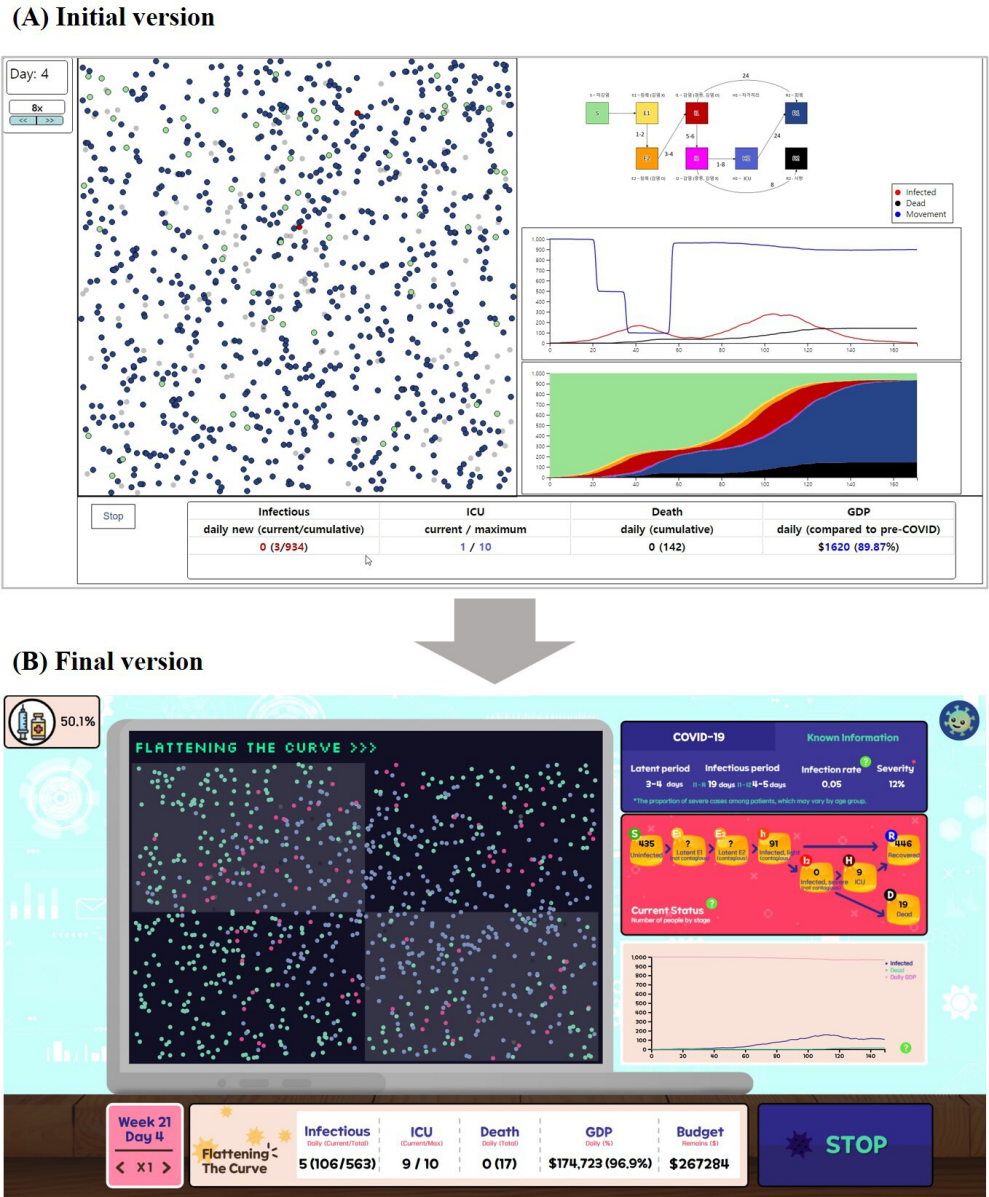
Results of overall design improvements. (A) The initial version. (B) The final version of the game. GDP: gross domestic product; ICU: intensive care unit.

**Figure 10.**
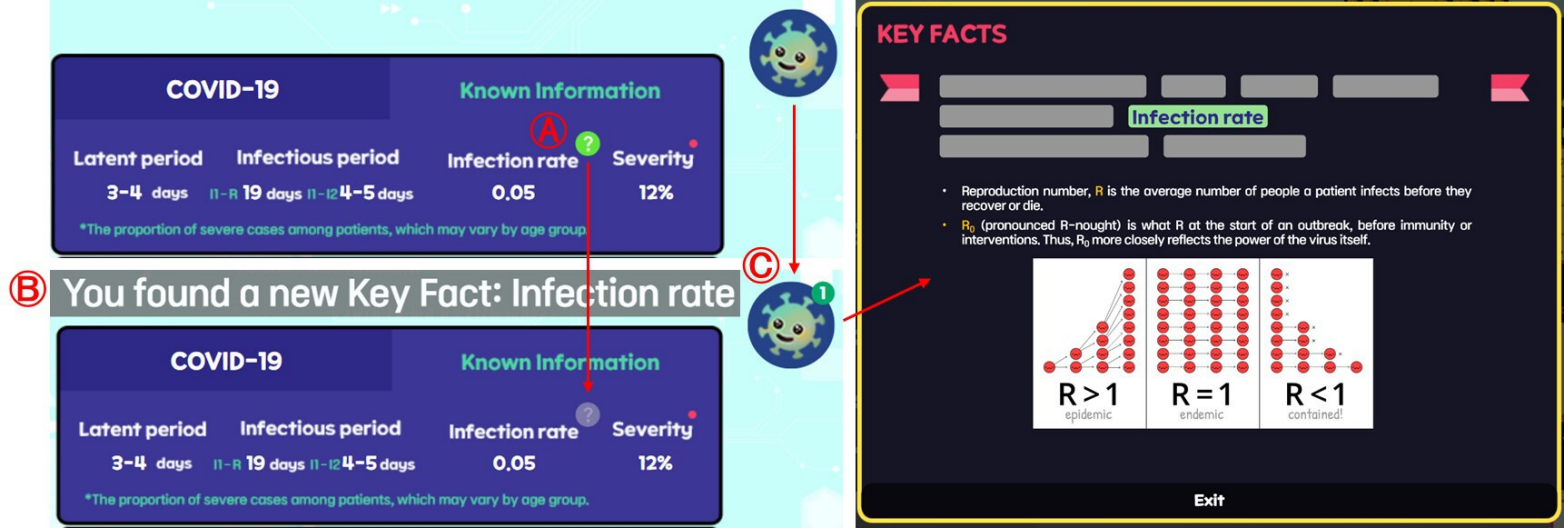
Overview of the Key Facts system. (A) When a player clicks a hint icon, (B) a message appears at the top, and (C) the Key Facts icon shows the count of newly discovered hints.

In terms of the user experience component, there were suggestions for improving the game’s UI and design, as well as incorporating in-game feedback and tutorials. Responding to participants’ opinions, we collaborated with design experts to completely rework the game design (Figure 9). To prevent monotony during repeated gameplay, a speed-up function was added, allowing users to accelerate simulation progress by up to 8 times. In response to feedback, budget notifications and pop-up alerts for budget shortages were implemented. Additionally, to assist new players in understanding and learning the game, we introduced a Key Facts tab providing essential information. Clicking on hint icons placed near elements requiring explanation adds the corresponding information to the Key Facts tab, allowing players to access explanations whenever needed (Figure 10).

### Game Evaluation

To assess if the game achieved its educational goals, we conducted surveys with the participants. The surveys measured the game’s educational impact and its influence on participants’ motivation to learn. In the earlier stage of PD, the first survey was conducted to measure participants’ satisfaction with the initial version of the game. The second survey was conducted at a later stage and focused on examining participants’ final evaluations of the game. Table 4 shows the descriptive statistics of the survey constructs.

Wilcoxon signed-rank tests were performed to examine the changes in participants’ satisfaction between the 2 surveys. Although participants reported higher satisfaction in the second survey, the difference was not statistically significant (*Z*=−1.890, *P*=.06).

Participants also gave an overall positive evaluation of the game-based learning experience, with average scores of 6.19 and 6.06 for perceived learning and enjoyment, respectively. The perceived usefulness and ease of use, variables to examine the technical acceptance of FTC, were also evaluated positively, with an average of 5.75 and 5.63, respectively.

**Table 4.** Descriptive statistics of survey items (N=16).

Construct		Rating range	Values, mean (SD)
**First survey**			
	Satisfaction	1‐4	2.94 (0.574)
**Second survey**			
	Satisfaction	1‐4	3.25 (0.447)
	Perceived learning	1‐7	6.19 (0.544)
	Enjoyment	1‐7	6.06 (0.755)
	Usefulness	1‐7	5.75 (0.848)
	Ease of use	1‐7	5.63 (0.717)

## Discussion

### Principal Findings

Since the COVID-19 outbreak began in 2019, there has been a proliferation of educational materials on various preventive measures for infectious diseases. However, there remains a need for educational resources that facilitate a fundamental understanding of infectious diseases and quarantine policies. This study presents the development and design process of a quarantine policy education game (“Flattening the Curve”). The game serves as a comprehensive educational simulation, allowing users to learn about the characteristics of infectious diseases, the stages of transmission, the necessity and effectiveness of quarantine policies, and the crucial balance between these policies and economic considerations. Ultimately, the game aims to enhance learners’ understanding of the concept of “flattening the curve.”

The application of the SERES framework played a pivotal role in achieving a scientifically sound and educationally effective game [25]. Drawing insights from prior research, the scientific and design foundations of the SERES framework were found to be effective in developing health-related serious games [17,66]. Building on these foundations, this study effectively developed various learning components within the game, such as the infection model, social distancing model, and economic model.

This study shows that both the SERES framework and the PD process can be used simultaneously and interactively during the serious game development process. The effectiveness of education in an e-learning environment is highly dependent on the learner’s motivation to learn [67], and an appropriate PD process during serious game development can lead to higher user satisfaction, which is essential for learner motivation [26,68]. The PD process was incorporated into the development of FTC to enhance the overall quality of the game and ensure learner engagement [69]. Participant feedback, categorized into the domains of learning, storytelling, gameplay, and user experience based on the DPE framework, guided the game’s improvement and detailed development direction [33].

As the survey results showed, the final game received positive evaluations from participants. The PD activities made the serious game more enjoyable and satisfactory and contributed to the enhancement of the game’s richness and user enjoyment.

### Limitations and Further Research

Despite our efforts to increase the game’s appeal to younger learners through design modifications guided by feedback from the PD process, this study has several limitations regarding its participants and evaluations. A comprehensive analysis of the game’s learning effects was not conducted. Although validating the effectiveness of educational games is crucial, this study did not measure quantifiable learning outcomes, such as changes in knowledge or attitudes. Due to the nature of the PD process, participants engaged in continuous gameplay and provided feedback throughout a semester, posing challenges in evaluating the knowledge acquisition effects solely through gameplay. Consequently, the current evaluation primarily focused on qualitative assessments of learners’ perceptions of the learning content.

Future research should target learners across various age groups, including children and adolescents. Measuring changes in knowledge related to infectious diseases and shifts in attitudes toward quarantine policies before and after gameplay can offer a more comprehensive evaluation of the learning effects of the FTC game. Additionally, we propose to evaluate the game with various game mechanics, such as difficulty and enjoyment. Investigating the relationship between game evaluations and learning effects will be instrumental in gaining a deeper understanding of the game’s impact on learners.

Although not specifically addressed in this study, we have experimentally developed various multiplayer modes to respond to participant feedback and assessed their impact on learner engagement. Consistent with prior research [70], participants found the game more engaging in multiplayer mode than in single-player mode. Subsequent research could delve into how multiplayer mode can make serious games better, highlighting the effects of different gameplay modes on learning outcomes and immersion. This underscores the importance of sustained research and development efforts in the serious game domain, particularly those focused on enhancing learning experiences through innovative gameplay features.

### Conclusions

“Flattening the Curve,” a serious game designed to promote understanding of epidemic models and quarantine policies, was successfully developed by applying the SERES framework and the PD process.

The developed game is now publicly accessible online, allowing anyone to play [71]. The game is not only designed to educate about COVID-19–related quarantine policies but also serves broader educational purposes related to various types of infectious diseases. The game’s source code is accessible to everyone, facilitating diverse educational applications and modifications to serve various research and educational objectives. The source code for the game is provided in a GitHub repository [72]. Resources are available under the license mentioned in the provided link. The authors welcome requests for additional information regarding the material presented in this paper.

## Supplementary material

10.2196/54968 Multimedia Appendix 1 Survey instrument details and reliability.

## Abbreviations

C: Critical
D: Dead
DPE: Design, Play, and Experience
E1: Exposed (not contagious)
E2: Exposed (contagious)
FTC: Flattening the Curve
GDP: gross domestic product
H: Hospitalized
I1: Infected, mild
I2: Infected, severe
ICU: intensive care unit
M: Mild
PD: participatory design
R: Recovered
S: Susceptible
SEIHR: Susceptible-Exposed-Infectious-Hospitalized-Recovered
SEIR: Susceptible-Exposed-Infectious-Recovered
SEMCR: Susceptible-Exposed-Mild-Critical-Recovered
SIR: Susceptible-Infectious-Recovered
UI: user interface
