# Supplementary material for: Serious Game Development for Public Health: Participatory Design Approach to COVID-19 Quarantine Policy Education
Source: JMIR Serious Games. 2024 Oct 15;12:e54968. doi: 10.2196/54968 (PMC11495237; doi:10.2196/54968)
Supplement: Multimedia Appendix 1 [file games-v12-e54968-s001.docx]

**Multimedia Appendix 1.** Survey instrument details and reliability.

| **Construct** | **Item** | **Questionnaire** | **Measurement** | **Source** | **Cronbach’s α** |
| --- | --- | --- | --- | --- | --- |
| Satisfaction | 1 | What is your overall satisfaction with the COVID-19 simulation? | 1: Very dissatisfied –  4: Very Satisfied | - |  |
| Perceived learning | 1 | Did you feel you were learning? | 1: Strongly disagree –  7: Strongly agree | [2] | 0.647 |
|  | 2 | Playing the game increased my understanding of science. |  |  |  |
|  | 3 | The game helped me learn. |  |  |  |
| Enjoyment | 1 | I enjoyed doing this activity very much. | 1: Strongly disagree –  7: Strongly agree | [1] | 0.845 |
|  | 2 | This activity was fun to do. |  |  |  |
|  | 3 | I thought this was a boring activity. ^a^ |  |  |  |
|  | 4 | This activity did not hold my attention at all. ^a^ |  |  |  |
|  | 5 | I would describe this activity as very interesting. |  |  |  |
|  | 6 | I thought this activity was quite enjoyable. |  |  |  |
| Usefulness | 1 | Learning using COVID simulation was effective. | 1: Strongly disagree –  7: Strongly agree | [3] | 0.764 |
|  | 2 | Learning using COVID simulation enhances learning efficiency. |  |  |  |
|  | 3 | COVID simulation encourages me to search for additional information on the topic of learning. |  |  |  |
| Ease of use | 1 | It is easy for me to handle the COVID simulation. | 1: Strongly disagree –  7: Strongly agree | [3] | 0.647 |
|  | 2 | I find it easy to study through the COVID simulation. |  | [3] |  |
|  | 3 | Overall, the COVID simulation is easy to use. |  | [4] |  |

^a^ Reverse coded item.

**References**

1. Center for Self-Determination Theory (CSDT). (n.d.). *Intrinsic Motivation Inventory (IMI)*. The Center for Self-Determination Theory (CSDT). Retrieved January 14, 2022 from https://selfdeterminationtheory.org/intrinsic-motivation-inventory/
2. Hamari, J., Shernoff, D. J., Rowe, E., Coller, B., Asbell-Clarke, J., & Edwards, T. (2016). Challenging games help students learn: An empirical study on engagement, flow and immersion in game-based learning. *Computers in Human Behavior*, *54*, 170-179. https://doi.org/10.1016/j.chb.2015.07.045
3. Jović, M., Kostic Stankovic, M., & Neskovic, E. (2017). Factors Affecting Students' Attitudes towards E-Learning. *Management: Journal of Sustainable Business and Management Solutions in Emerging Economies*, *22*(2), 73. https://doi.org/10.7595/management.fon.2017.0016
4. Lee, M.-C. (2010). Explaining and predicting users’ continuance intention toward e-learning: An extension of the expectation–confirmation model. *Computers & Education*, *54*(2), 506-516. https://doi.org/10.1016/j.compedu.2009.09.002
